# Improved nonparametric survival prediction using CoxPH, Random Survival Forest & DeepHit Neural Network

**DOI:** 10.1186/s12911-024-02525-z

**Published:** 2024-05-07

**Authors:** Naseem Asghar, Umair Khalil, Basheer Ahmad, Huda M. Alshanbari, Muhammad Hamraz, Bakhtiyar Ahmad, Dost Muhammad Khan

**Affiliations:** 1 Department of Statistics, Abdul Wali Khan University Mardan, Mardan, KP Pakistan; 2 Department of Statistics, University of Swat, Swat, KP Pakistan; 3 Department of Mathematical Sciences, College of Science, Princess Nourah bint Abdulrahman University, P.O.Box 84428, Riyadh, 11671 Saudi Arabia; 4 Higher Education Department, Kabul, Afghanistan

**Keywords:** CoxPH, DeepHit Neural Network, Machine learning, Survival analysis, High-dimensional data, Feature selection, Random Survival Forest, CoxBoost, LASSO, SCAD

## Abstract

In recent times, time-to-event data such as time to failure or death is routinely collected alongside high-throughput covariates. These high-dimensional bioinformatics data often challenge classical survival models, which are either infeasible to fit or produce low prediction accuracy due to overfitting. To address this issue, the focus has shifted towards introducing a novel approaches for feature selection and survival prediction. In this article, we propose a new hybrid feature selection approach that handles high-dimensional bioinformatics datasets for improved survival prediction. This study explores the efficacy of four distinct variable selection techniques: LASSO, RSF-vs, SCAD, and CoxBoost, in the context of non-parametric biomedical survival prediction. Leveraging these methods, we conducted comprehensive variable selection processes. Subsequently, survival analysis models—specifically CoxPH, RSF, and DeepHit NN—were employed to construct predictive models based on the selected variables. Furthermore, we introduce a novel approach wherein only variables consistently selected by a majority of the aforementioned feature selection techniques are considered. This innovative strategy, referred to as the proposed method, aims to enhance the reliability and robustness of variable selection, subsequently improving the predictive performance of the survival analysis models. To evaluate the effectiveness of the proposed method, we compare the performance of the proposed approach with the existing LASSO, RSF-vs, SCAD, and CoxBoost techniques using various performance metrics including integrated brier score (IBS), concordance index (C-Index) and integrated absolute error (IAE) for numerous high-dimensional survival datasets. The real data applications reveal that the proposed method outperforms the competing methods in terms of survival prediction accuracy.

## Introduction

Survival data analysis is a statistical subfield focused on studying the duration until a specific event occurs, such as the time until death in living organisms or the time until failure in mechanical systems. It aims to answer questions like the proportion of a population expected to survive beyond a given time point, the rate at which those who survive will experience the event, and how the likelihood of survival varies with different conditions or characteristics. The objectives of survival analysis are to identify relationships between risk variables and occurrences, to explain the likelihood of an event occurring by a certain period, or to forecast survival times based on informative characteristics. Survival outcomes are generally referred to as events or results related to the duration of time that an individual or entity survives or remains in a particular state before a specific event occurs such as death, relapse, or a specific health condition developing.

In recent decades, there has been an increasing focus on devising methods for selecting relevant features in time-to-event data. This heightened interest is driven by the availability of extensive datasets and the recognition that data sparsity may exist. When we refer to sparsity, we mean that certain features in the dataset may not have any relevance to the outcome of interest, and variable selection becomes the preferred approach to address this. A number of feature selection (FS) techniques have been developed in literature, each working for the same objective function that is to reduce dimension of the data but using a different mechanism to reach the goal. FS algorithms available are numerous but it is important to mention the saying ‘one-size-fits-all’ type algorithm does not exists in reality. In such cases, margin of error always exists which needs to be minimized. This motivates researchers to improve already existing algorithm/technique or come up with a fresh idea to do the same task with minimum error. With this in mind, we tried to introduce a hybrid type FS algorithm for survival analysis whose working mechanism is explained in the next section.

### Related work

Numerous methods for selecting variables in survival data have been devised over time. To provide a concise overview of the existing body of work and to gain insight into the functionality of each algorithm in this context, a brief literature review is presented below.

Tibshirani [1] extended the Least Absolute Shrinkage and Selection Operator (LASSO) method to the Cox model that was initially introduced for linear regression. In this approach, an L1-norm penalty term is added to the loss function. The coefficients (β) are then estimated via maximization of the partial likelihood function while observing the constraints $\sum {|\beta }_{j}|\le s$ where ‘s’ is a user-defined non-negative value. By effectively shrinking the coefficients of less significant and superfluous variables to zero, this constraint lowers the complexity of the model.

The Adaptive LASSO for the Cox proportional hazards model was developed by Zhang and Lu [2] to improve the estimator's characteristics and make it work with common techniques. In order to achieve equilibrium, this strategy gives larger weights to small coefficients and smaller weights to large coefficients. Global optimizers are certain to exist because of the convex shape of the penalty term. The adaptively weighted $L1$ penalty of the form $\lambda {\sum }_{k=1}^{p}|\frac{{\beta }_{k}}{{\widetilde{\beta }}_{k}}|$ where ${\widetilde{\beta }}_{k}=({\widetilde{\beta }}_{1}, {\widetilde{\beta }}_{2}, {\widetilde{\beta }}_{3},\dots , {\widetilde{\beta }}_{p})$ known as Adaptive Lasso penalty maximized partial likelihood.

In certain situations, LASSO has shown limitations. For instance, it tended to limit the number of features based on a systematic relationship with the number of samples in the dataset. Additionally, when dealing with highly correlated predictors, it often selected only one feature among them. To overcome these challenges, alternative methods have been introduced, one of which is Elastic Net. This method involves incorporating a combination of L1 and L2-norm penalties into the regression coefficient estimation process. The constraint of the form $\lambda \left(\left(1-\alpha \right)\sum_{j=1}^{p}\left|{\beta }_{j}\right|+\alpha \sum_{j}^{p}{\beta }_{j}^{2}\right)$ is added to the partial log-likelihood function while maximizing it during the estimation process [3].

Cross-validation was used to estimate the two parameters, regularization parameter $\lambda$ and mixing parameter α. Later, it was suggested to just estimate $\lambda$ by cross-validation using a fix α = 0.5.

Du et al. [4] introduced a method for variable selection in the Cox Proportional Hazards model with semi-parametric relative risk. They initially divided the model into two components: parametric and non-parametric. For the non-parametric component, they employed a smoothing spline ANOVA model to estimate risk, and variable selection was performed using the Kullback–Leibler geometry method. In contrast, for the parametric component, risk estimation was conducted using the Penalized Profile Partial Likelihood approach. Variable selection in this part was achieved by applying a concave penalty, either SCAD (Smoothly Clipped Absolute Deviation) or an Adaptive LASSO penalty. It is advisable to incorporate discrete covariates into the parametric component and continuous covariates into the non-parametric component of the model. Nevertheless, if the estimation process indicates that certain continuous covariate effects can be suitably represented by specific parametric forms, like linear relationships, these covariates can be transferred to the parametric section, and a fresh model can be developed accordingly.

Li and Luan [5] introduced a boosting algorithm utilizing smoothing splines, which constructs a series of smoothing spline models and amalgamates them into a final model. This iterative algorithm modifies the hazard function at each step to rectify errors from prior models. In a comparative study against traditional proportional hazards models, Li and Luan demonstrated the superiority of their boosting algorithm, particularly in analyzing high-dimensional microarray data from a breast cancer study. Their research underscores the efficacy of the boosting algorithm with smoothing splines for survival analysis in high-dimensional datasets and its capacity to handle complex covariate structures in survival analysis.

Morris et al. [6] devised a feature selection technique tailored for stratified Cox models using gradient boosting. This method is intended for situations where the assumption of proportional hazards is not met. To mitigate confounding effects, they introduced a stratification process, resulting in a stratified proportional hazards model. In their algorithm, variables are chosen based on their capacity to maximize the first partial derivative of the likelihood function. This iterative process updates β coefficients estimates while monitoring the risk of overfitting, with the number of iterations determined through various options like Bayesian Information Criterion (BIC), a predefined number of variables for selection, changes in likelihood, or k-fold cross-validation. Additionally, they developed an R package called 'SurvBoost' to implement this method and conducted a simulation study comparing its accuracy and runtime with the existing 'mBoost' R package.

He et al. [7] introduced an algorithm that applies gradient boosting to the data while considering only one component of β, aiming to improve computational feasibility. The fundamental concept behind this algorithm involves modifying certain aspects of existing variable selection techniques designed for high-dimensional survival data. Although component-wise boosting algorithms were already present in the literature, the authors made specific adaptations to enhance its computational efficiency, drawing inspiration from the Minimization-Maximization (MM) algorithm by Hunter and Lange [8].

When conducting FS on randomly sampled observations, the objective is to determine the probability that features are included in the model. This approach is known as stability selection [9]. Additionally, the authors proposed a stability selection boosting procedure based on random permutations, which follows the concept introduced by Tusher et al. [10] in the context of the Significance Analysis of Microarrays (SAM). This modification resulted in a reduced false discovery rate for their variable selection algorithm.

Ishwaran et al. [11] introduced a novel approach in survival tree analysis by introducing a dimensionless ordered statistic known as the 'minimal depth of maximal subtree.' This statistic served as a measure of variable predictiveness within survival trees. Unlike the traditional method of calculating Variable Importance (VIMP) in random forests using permutation, they opted for the minimal depth statistic. This statistic assesses the importance of variables based on their proximity to the root node in a tree, specifically in relation to the root of the nearest maximal subtree. Their variable selection procedure comprises three main steps. Initially, they randomly select covariates for the model and identify the most crucial ones using minimal depth. An initial model is then constructed with these chosen covariates. This process is iterated a predefined number of times, incorporating additional variables into the initial model based on minimal depth criteria until the joint VIMP of nested models stabilizes. This entire process is repeated several times, and the covariates that consistently appear in models larger than the average size are ultimately selected.

Pang et al. [12] introduced a gene selection method that employs an iterative feature elimination procedure within the framework of random survival forest (RSF). Their approach begins by fitting an RSF model to the dataset containing the complete set of covariates and ranking all covariates based on their Variable Importance (VIMP) scores, which are calculated using a permutation-based method. The top variables, typically around 80%, are retained, and their out-of-bag errors are computed. This process is repeated iteratively until only two covariates remain in the model. The objective is to identify the set of covariates with the minimum number required to maintain an out-of-bag error rate within 1 standard error. This methodology effectively takes into account the multivariate correlations among variables. Experimental results on real high-dimensional microarray datasets with survival outcomes demonstrated that this approach excels in identifying a compact set of genes while preserving predictive accuracy for survival.

Mbogning and Broet [13] introduced a variable selection approach tailored for survival data, which relies on a Topological Index derived from permutation methods. Their methodology begins with the construction of a bagging survival forest on the training data. The importance score, utilized as a criterion for node splitting or determining tree depth during forest construction, serves as the basis for calculating an importance score. These importance scores are denoted as ${HS}_{j}(j = \mathrm{1,2},3,\dots ,p)$ ***.*** Subsequently, another bagging survival forest is created, but this time, the importance of variables is computed based on permuted data. This process is iterated a specified number of times. Another list of scores are generated in ${HS}_{j}^{*} (j = \mathrm{1,2},3,\dots ,p)$. *P*-values are calculated for all competing variables ${X}_{j}$ using ${P}_{j}=\frac{1}{Q}\sum_{q=1}^{Q}I\{{HS}_{jq}^{0}>{HS}_{j}\}$. Given a global level α, variables which satisfies the conditions that ${p}_{j}<\frac{a}{m}$ are selected according to a Bonferroni procedure for multiple comparisons.

Indeed the above mentioned techniques are useful for variable selection, their application in this study is however limited for identifying discriminative features in high-dimensional survival datasets. Therefore, we have proposed a novel tools for variable/feature selection due their ability in handling high-dimensional survival data which is important for better predictions.

## Methods and material

### Datasets and data processing

In this section, we explore the datasets considered in this research study. A total of 11 survival datasets were used and their detail description along with their source is presented in Table 1. While selecting the datasets for the current research study related to feature selection in survival analysis, it was ensured that the benchmark datasets are high-dimensional in nature.

**Table 1:** Description of the Benchmark high-dimensional survival datasets

Serial No	Name of Dataset used in this study	No. of Samples	No. of Features	Source
1	**Breast:** Contains clinical and genomic data of 614 early breast cancer patients	614	1692	[14]
2	**WPBC:** Stands for Wisconsin Prognostic Breast Cancer. Contains clinical data and follow-up information for patient with breast cancer	198	34	[15]
3	**VDV**: van de Vijver Microarray Breast Cancer dataset	78	4707	[16]
4	**Heart FD:** Heart Failure Dataset collected for research purpose by a group of students at Faisalabad, Pakistan	299	13	[17]
5	**MNO:** Short for Melanoma Nanostring dataset	45	206	[18]
6	**GE1:** A gene expression data measured by DNA microarrays from breast tumor patients	115	553	[19]
7	**GE2:** A gene expression dataset comprising patients diagnosed with primary breast carcinomas, all of whom had either stage I or III breast cancer and were under 53 years of age	116	4753	[20]
8	**GE3:** Gene expression data collected from peripheral-blood and bone marrow samples of patients diagnosed with acute myeloid leukemia (AML)	116	6288	[19]
9	**DLBCL:** The dataset consists of gene expression and survival data from a cohort of 240 patients diagnosed with diffuse large-B-cell lymphoma	240	7399	[21]
10	**Bone M:** Data pertaining to pediatric patients suffering from various hematologic diseases who underwent unrelated donor hematopoietic stem cell transplantation (UD HSCT) without manipulation	187	37	[22]
11	**NKI:** A subset dataset based on top varying genes from gene expression dataset	272	1567	[23]

### The Cox PH model

The Cox Proportional Hazard Model (CPHM) is a semi-parametric method used in survival analysis. It defines the hazard function $'h(t)'$ as the sum of two components: a baseline hazard $'h_0(t)'$ that depends solely on time 't,' and a component related to covariates. The mathematical form of the CPHM is as follows:

1 $$h\left(t\right)={h}_{0}\left(t\right)\times {\text{exp}}\left({\beta }_{1}{x}_{p1}+{\beta }_{2}{x}_{2}+{\beta }_{3}{x}_{p}+\dots +{\beta }_{i}{x}_{i}\right),={h}_{0}\left(t\right) \times {{\text{e}}}^{\left({\beta }{\prime}{x}_{i}\right)}$$

Here in this equation, ${h}_{0}(t)$ is a time-dependent component that is not influenced by covariates, while ${e}^{{\beta }{\prime}{x}_{i}}$ represents the covariate-related component, which does not depend on time’t’. It's important to note that there is no constant term “${\beta }_{0}$” in the regression coefficients. This absence of a constant term is because it can be absorbed or canceled out by the baseline hazard function, essentially being a part of the hazard function itself [24].

#### Feature selection methods

With the introduction of new technologies in past few decades, we are able to access such information about a subject under study which one had never before. Having more information about samples/subjects on the other hand can create a situation called the curse of dimensionality. Especially in the case when number of features are substantially greater than the number of observations. This create several problems during the analysis and processing of the data, such as increase in computational cost, noise and redundancy, the problem of overfitting and poor generalization of performance on unseen data [25]. There are three common types of feature selection methods namely filter, wrapper and embedded methods.

##### Filter methods

This consists of feature selection techniques that ranks the features in dataset ahead of running a learning algorithm and selects features in the model based on a pre-specified criteria in connection with the statistical measure being used for ranking purpose. This set of methods are less time-consuming and inexpensive in nature as they are done as a pre-processing step.

##### Wrapper methods

Wrapper methodology consists of techniques where subsets of features are made, model is trained on each subset and comparison is made for each subset in terms of performance metrics. Features are added or removed from the subsets until a pre-defined number of features and performance measure is obtained. The subset which yields a pre-defined model output is considered as a final set of features for modelling the data further.

##### Embedded methods

This method combines the core properties of both, the filter and wrapper methods. It is named as embedded because the feature selection technique is blended as a part of the actual learning algorithm. It is a less time-consuming, inexpensive and more accurate than aforementioned methods. The methods employed in this study for comparison fall under this category. In the following section, we provide a brief overview of the variable selection methods utilized in this study.

### Least Absolute Shrinkage and Selection Operator – LASSO

Least Absolute Shrinkage and Selection Operator, or LASSO, is a method that penalises variables. It introduces an L1-type penalty term λ||β|| to the coefficients of the Cox regression [1]. This penalty can effectively reduce some coefficients to zero, leading to a reduction in the model's size while maintaining its parsimony. The parameter λ plays a crucial role in determining the number of variables selected in the model. A larger λ value leads to more coefficients being reduced to zero, resulting in a model with fewer features. Conversely, reducing the λ value increases the number of features included in the model when compared to a higher λ value.

In a survival context, the triplet $\{\left({Y}_{i}, {\delta }_{i}, {X}_{i}\right), i=\mathrm{1,2},3, \dots , n\}$ is used to represent the observed data. Where ${Y}_{i}={\text{min}}({T}_{i}, {C}_{i})$ is the observed survival time, taking minimum of either observed event time “${T}_{i}$” or censoring time “${C}_{i}$”. ${\delta }_{i}$ is the censoring indicator, ${\delta }_{i}=I({{T}_{i}\le C}_{i})=1$ when actual event of interest is observed and is 0 otherwise. And ${X}_{i}={({x}_{1}, {x}_{2}, {x}_{3},\dots ,{x}_{p})}^{t}$ is the matrix of ‘p’ predictor variables for each subject in the dataset. To model the survival data, we consider semi-parametric cox proportional hazards model: $h\left(t|x\right)={h}_{0}(t){\text{exp}}(\sum_{j=1}^{p}{B}_{j}{X}_{j})$.

The partial likelihood for cox model is given as: ${L}_{n}\left(\beta \right)={\prod }_{i\in D}\frac{{\text{exp}}({X}_{i}^{t}\beta )}{\sum_{l\in {R}_{i}}{\text{exp}}({X}_{l}^{t}\beta )}$.

Where D is the set of indices for observed events, and ${R}_{i}$ are observations at risk at time ${Y}_{i}$. Now, a function known as log partial likelihood i.e., ${l}_{n}\left(\beta \right)={\text{log}}\left\{{L}_{n}\left(\beta \right)\right\}/n$ with a penalty term, specifically called lasso penalty i.e., ${p}_{\lambda }\left(\beta \right)=\lambda \sum_{j=1}^{p}|{\beta }_{j}|$ applied on the coefficients β, when minimized, results in the sparsity, hence variable selection.

Mathematically: $$g\left(\beta\right)=l_n\left(\beta\right)+\rho_\lambda\left(\beta\right)=,g\left(\beta\right)=\frac{\log\left\{\left(\beta\right)\right\}}n+\lambda{\textstyle\sum_{j=1}^p}\left|\beta_j\right|,\left(\beta\right)=-l_n\left(\beta\right)+\lambda{\textstyle\sum_{j=1}^p}\left|\beta_j\right|.$$

Where λ is a non-negative tuning parameter taking on any positive value and controls the amount of variables selected in the final model. The lasso penalty $\lambda \sum_{j=1}^{p}|{\beta }_{j}|$ is singular at point ${\beta }_{j}=0$ and is therefore able to eliminate the redundant variables from the model and keep the relevant ones only.

#### Random Survival Forest’s Variable Selection – RSF-vs

A useful tool for variable selection is the Random Survival Forest (RSF), which is an extension of the random forest approach for survival data [26]. RSF builds trees in a similar way as conventional random forests. Following the random selection of B bootstraps at random from the data, a tree is created on each bootstrap sample. A cumulative hazard function (CHF) is produced by averaging the predictions made by these trees. The RSF then offer two option for feature selection: the variable hunting algorithm (RSF-VH) and minimal depth. The minimal depth approach for FS is advised when the ratio of the number of features (p) to the number of samples (n) is less than ten that is p/n < 10. However, when p/n > 10, the RSF-VH approach for FS is preferred. When splitting of a node is carried out, the minimal depth is used to rank the features according to their distance from the root node in the tree. Shorter paths between variables and the root node are regarded as having greater predictive power in the model. More detailed information on minimal depth of the maximal subtree can be found in the work by Ishwaran et al. [11]. In RSF-VH, an initial model is constructed using covariates according to a predetermined minimal depth threshold value. Additional covariates are gradually incorporated into the initial model based on their minimal depth rankings until the joint variable importance (VIMP) for the resulting nested models stabilizes. This process is typically repeated multiple times, often 50 repetitions, and the variables that are frequently selected in the models are included in the final model.

#### Smoothly Clipped Absolute Deviation – SCAD

SCAD was proposed by Fan and Li [27] as an improved alternative to LASSO for penalizing the regression coefficients. Some studies [27–29] showed that LASSO can come up with biased results for coefficients with larger values, while working fine for the coefficients with relatively smaller values. This led the researchers to introduce another penalty term known as non-concave SCAD-penalty.

The penalty function is rather defined primarily by its first derivative which is given as:

2 $$\begin{array}{cc}\overset{\mathit,}p\left(\beta\right)=\lambda\{I\left(\beta\leq\lambda\right)+\frac{\left(a\lambda-\beta\right)+}{\left(a-1\right)\lambda}I\left(\beta>\lambda\right)\}&\mathrm f\mathrm o\mathrm r\,\mathrm s\mathrm o\mathrm m\mathrm e\,a\,>\,2\,\mathrm a\mathrm n\mathrm d\,\beta>0.\end{array}$$

This penalty term contains two tuning parameters that are, $\lambda$ and $a$ whose values could be obtained by some criteria such as cross validation based on a grid search for the best pair of ($\lambda , a$) values. But this could be computationally very expensive too. Thus, based on Bayesian statistical point of view and practical simulation studies, Fan and Li [27] suggests using, $a=3.7$.

#### Boosting Algorithm for Variable Selection – CoxBoost

Boosting, originally developed in machine learning for classification and regression problems [30–32]. It is basically an ensemble technique which runs iteratively to combine the predictions of many weak models into one strong model. With time, boosting algorithms started getting notable attention, and were later on extended to statistical field, operating in many statistical problems including regression and survival analysis.

We utilized the boosting algorithm known as ‘CoxBoost’ to perform feature selection making use of the cox regression model. For this, we used an R package ‘mboost’ [33] which performs a model based boosting using the built-in function ‘coxPH’ to be specified in argument ‘family’. Using this argument, we are about to use negative partial log-likelihood as a loss function L(y, F(X)) and OLS estimator as the base learner. The complete boosting algorithm for model fitting as well as feature selection context completes in these five steps.

1. Initialize $$\widehat{\beta }=\left(0,\dots ,0\right);$$
2. Compute the negative gradient vector: $u^{(i)}=\delta^{(i)}-\sum_{l\in R^{\left(i\right)}}\delta^{(i)}\frac{\text{exp}\{X^{(l)T}\widehat{\beta\}}}{\sum\{X^{\left(l\right)T}\widehat\beta\}}$
3. Compute the possible updates to the gradient vector by fitting least square estimator, $\overset\frown{b_j}={(X_j^TX_j)}^{-1}X_j^Tu;$
4. Select the best update, $j^\ast={argmin}_j{\textstyle\sum_{i=1}^n}\left(u^{\left(i\right)}-X_j^{\left(i\right)}{\widehat b}_j\right)^2;$
5. Update the estimate, ${\widehat{\beta }}_{j*}={\widehat{\beta }}_{j*}+v{\widehat{b}}_{j*}.$

The steps 2–5 are repeated m_stop_ number of times, which plays a crucial role in both, feature selection and prediction models. In feature selection, increasing this will increase the number of features selected and vice versa. This can create problems of overfitting and irrelevant variable selection in the model in case of larger value while we may miss important predictor variables if this value is kept low [34]. To cope with this issue, a tenfold cross validation is used to select the optimal value for m_stop_ – which is the number of boosting steps – and do the feature selection using that optimal m_stop_ value.

#### Machine learning models

Time-to-event data can be analyzed for making predictions about survival time and estimate the survival probability at a specific estimated survival time, using both traditional statistical methods and machine learning models. Although, they both share this common goal of making predictions of the survival time and estimating the survival probabilities, yet the focus of both methods is on different objectives. Traditional methods mainly focuses on distributions of event times and statistical properties of estimation of parameters, whereas machine learning models combines the power of traditional methods along with machine learning techniques to make better predictions of occurrence of event at a given time [35].

There are three types of statistical methods commonly used in the context of survival analysis that are Parametric, Semi-parametric and Non-parametric. In this study, the semi-parametric approach, i.e., the Cox Proportional Hazards Model, is employed for predictions, along with two other machine learning models: Random Survival Forest and DeepHit Neural Network. These models are used for modeling time-to-event data and are discussed in the following section.

#### Random Survival Forest (RSF)

RSF is an ensemble method usually categorized under advanced machine learning techniques which is basically an extension of random forests approach to tackle survival information [36, 37].

The forest is grown in the same manner as in a usual random forest i.e.

i. Randomly select B samples of the same size as the original dataset, allowing for replacement. Any samples not chosen are considered out-of-bag (OOB).
ii. Construct a survival tree for each of the B samples selected in the first step.
  a At each tree node, randomly choose a subset of predictor variables and determine the best predictor and splitting value that yield two subsets (referred to as daughter nodes) with the maximum difference in the objective function.
  b Repeatedly apply the above step recursively to each daughter node until a specified stopping criterion is met.
iii. Calculate the cumulative hazard function (CHF) for each tree and then compute the average CHF across all B trees to create the ensemble CHF.
iv. Assess the prediction error of the ensemble CHF using solely the OOB data.

Since the CHF and survival function S(t) are related, the RSF also gives us estimates of survival function which we can further use for predictions using the test set of the data and calculate our performance evaluation metrics.

#### DeepHit neural network

DeepHit is a deep learning model designed for survival analysis, capable of simultaneously addressing single-cause and competing risks scenarios. It utilizes a network structure comprising a shared sub-network and several cause-specific sub-networks. DeepHit's training process involves a loss function that leverages both survival times and relative risks. This model is proficient in capturing non-linear and non-proportional relationships between covariates and risk factors. Further, it is a discrete-time survival model, means that survival times are discretized into either equidistant (equally spaced) or quantiles intervals [38]. The DeepHit method was the first to implement neural networks to the discrete-time likelihood for survival data. A more detailed reading on how discretization process works in survival context and its further extensions can be obtained from a study conducted by Kvamme, H. and Borgan, Ø., 2021 [39].

In this study, we implemented the DeepHit NN using R package ‘survivalmodels’ [40] which makes it happen using R package ‘reticulate’ [41]. Where Reticulate is a popular R package creating a Python environment in R software so that one can use Python packages and functions inside R [42]. No hyper parameter tuning was applied, rather the default parameters were used for training the neural network.

#### Proposed model architecture

In this paper, Feature selection methods described some well-known feature selection techniques for survival studies, briefly explaining their algorithms to separate and select only important features to the response variable. In practice, it is usually observed that employing many FS methods to the same dataset, it is not guaranteed that all techniques agree upon the same set of features. It is due to the mechanism a FS technique uses to give relevant importance to features. For instance, RSF-vs uses a dimensionless order statistic called minimal depth of maximal subtree which shows the predictive power of a variable in a survival tree. RSF-vs ranks features based on minimal depth criteria and selects the top-most from the list. In contrast, LASSO and SCAD implies their respective penalties on the regression coefficients in order to contract redundant variables’ coefficients to as low as zero. Although, the mechanism each method uses are way different than each other, yet the objective function is the same for all, so it is expected from each to agree upon other’s selection as much as possible, off course if not hundred percent.

In this work, we aimed at exploiting and capitalizing four different feature selection techniques that uses contrasting mechanism to obtain the objective function. Intuitively, the variables which are important and non-redundant in reality is believed to have higher chances of being chosen by most of the algorithms. This led to propose a novel feature selection method for survival data.

The proposed method is a three-step procedure for feature selection, constituting a hybrid method that selects the most informative or discriminative features on which the majority of the feature selection techniques agree. The method is explained as follows:

i. Utilize each of the four feature selection techniques—LASSO, RSF-vs, SCAD, and CoxBoost—individually to obtain four feature sets, one for each of the four FS techniques employed.
ii. After comparison, create a new set that only includes those features chosen for at least three of the four sets in step 1.
iii. The final set of features is determined by selecting the set of characteristics that satisfy the criteria in step 2. These features will then go through additional processes, like fitting a survival model utilising predictive machine learning models and assessing their predictive accuracy.

The suggested alg0rithm is described mathematically as follows:

- Let FS 1​, FS 2​, FS 3​, and FS 4​ stand for the sets of feature that were 0btained by using the LASSO, RSF-vs, SCAD, and C0xB00st methods, respectively.
- Then, the common features that has agreement or the set of intersection of these feature, represented as FS intersection​, is determined by:
- $${FS}_{\,intersection}=\left\{f\in F/FS_1\cap FS_2\cap FS_3\cap FS_4\right\}$$
- Next, the features that show up in at least three of the four feature sets are then filtered out as:
  $$\begin{array}{c}\mathrm{FS}\;\mathrm{final}=\left\{\mathrm f\mid\;\mathrm f\;\in\;\mathrm{FS\,intersection},\mathrm{count}(\mathrm f)\;\geq\;3\right\}\\\mathrm{Here}\;\mathrm{count}\;\left(\mathrm f\right)=\mid\left\{{\mathrm{FS}}_{\mathrm i}\mid\mathrm f\mathit\;\mathit\in{\mathrm{FS}}_{\mathrm i}\right\}\mid\end{array}$$

where *count(f)* is the number of times feature *f* appears throughout the four feature sets.

The final collection of attributes, denoted by the set *FS*_final​_, will be used for additional analysis, including the fitting and performance evaluation of survival models.

The primary goal of this suggested approach is to create a feature selection pr0cess for survival predictions that is both simple to comprehend and apply. The 0bjective of this study is to develop an appr0ach that is both efficient and intuitive by combining well-known feature selection techniques with a simple selection criterron based 0n their agreement across these techniques. Despite its simplicity, the prop0sed method has shown promising results in 0ur experiments, as evidenced by its performance in survival prediction tasks compared to existing techniques.

The algorithm of the proposed hybrid feature selection method is given below.

**Figure:**
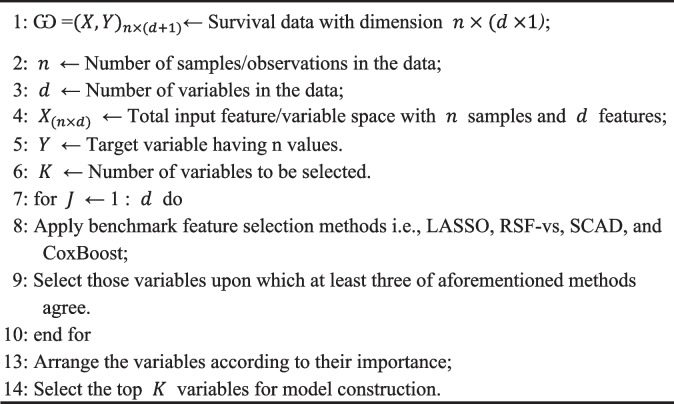
**Algorithm 1:** Pseudo code of the proposed method

**Fig. 1:**
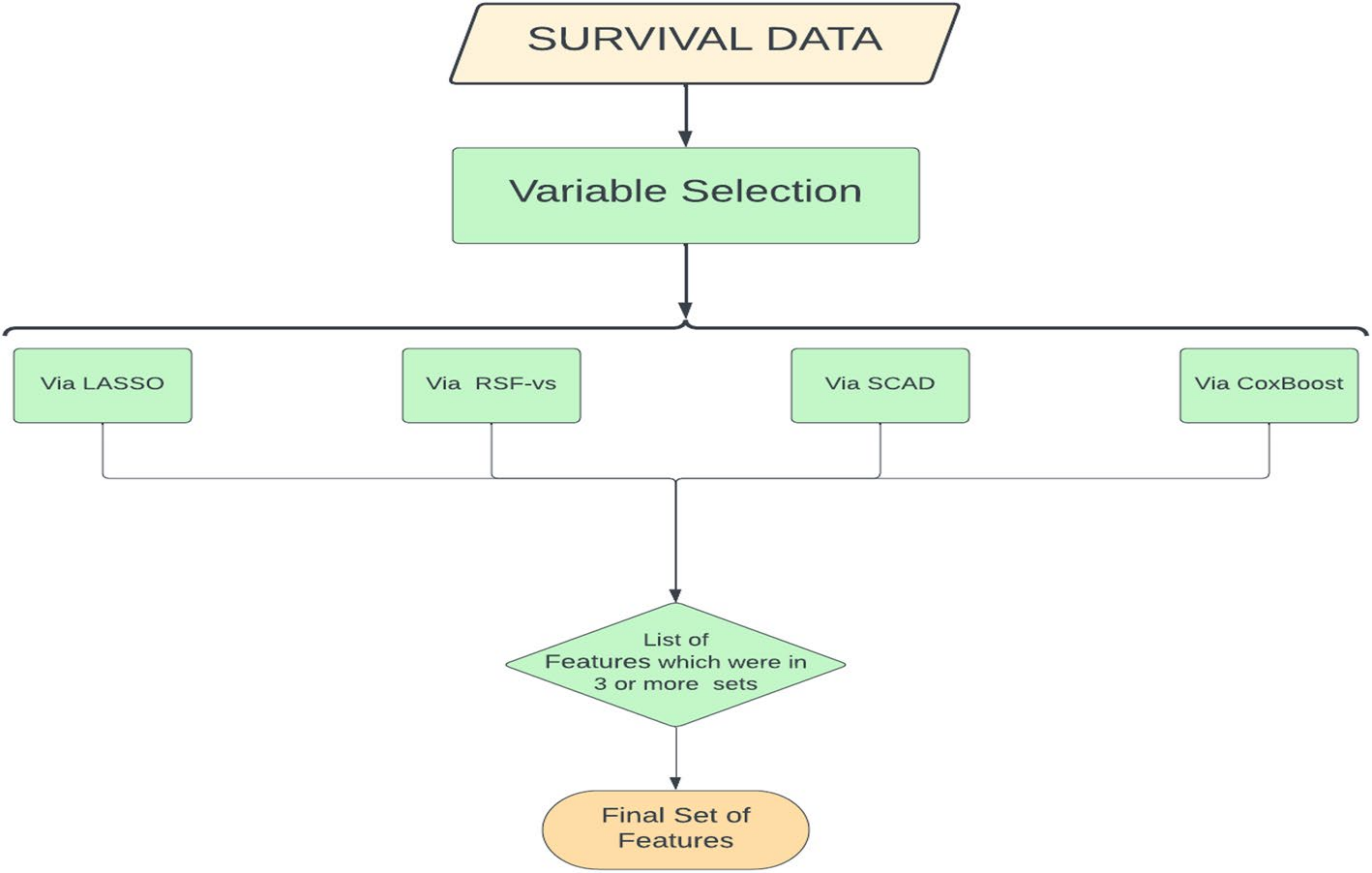
A flowchart of the proposed feature selection method for survival analysis

The following flowchart shows the basic outline of the proposed method in a graphical way in Fig. 1.

## Performance evaluation metrics

To evaluate model performance, researchers have access to various evaluation metrics, allowing them to choose the most appropriate ones for their specific problem. For survival models, common metrics include the Concordance index [43], C-statistic (a modified version of C-index suitable for models with high censoring rates) [44], Brier score, integrated Brier score [45], integrated square error (ISE) [46], and others. In this study, we chose to assess the competitive models using Integrated Brier Scores (IBS), the C-Index, Integrated Absolute Error (IAE), and Integrated Square Error (ISE). These methods are briefly explained as follow:

i. Integrated Brier Score (IBS)

The Brier score, initially introduced by Brier in 1950 [45] to assess the accuracy of weather forecasts, was later adapted to evaluate the performance of survival models that incorporate censored observations [47]. The Brier score varies with time. In the absence of censoring, the Brier score can be expressed as:

3 $$\begin{array}{cc}BS\left(t\right)=\frac1N\sum\limits_{i=1\dots N}\left\{\begin{array}{c}(0-\widehat S{(t/z_i))}^2\\(1-{\widehat S(t/z_i))}^2\end{array}\right.&\begin{array}{c}\text{If}\,t_i\leq t\\\text{I}f\,t_i\geq t\end{array}\end{array}$$

In scenarios involving censoring, a weighted version of the formula is used to accommodate censoring. Specifically, BS(t) is divided by $1/\widehat{G}({t}_{i})$ when censoring occurs before time ‘t’ and it is divided by $1/\widehat{G}(t)$ when censoring occurs after time 't.' Observations that are censored before time 't' are not included in the Brier score calculation. The formula for calculating Brier score in the presence of censoring is as follows:

4 $$\begin{array}{ll}BS\left(t\right)=\frac1N\sum\left\{\begin{array}{c}\frac{(0-\widehat S{(t/z_i))}^2}{\widehat G(t_i)}\\\frac{(1-{\widehat S((t/z_i))}^2}{\widehat G\left(t\right)}\\\\0\end{array}\right.&\begin{array}{l}\text{If}\,t_i\leq t,\delta_i=1\\\begin{array}{l}\text{If}\,t_i>t\\\mathrm{If}\,t_i=t,\delta_i=0\end{array}\end{array}\end{array}$$

A Brier score approaching 0 signifies superior predictive performance, while a score nearing 1 suggests poorer performance. The Integrated Brier Score (IBS) is derived by integrating the Brier score across all available time intervals, denoted as ${t}_{min}\le t\le {t}_{max}$. Mathematically, this can be expressed as follows:

5 $$IBS= \frac{1}{{\text{max}}\left({t}_{i}\right)}{\int }_{0}^{{\text{max}}\left({t}_{i}\right)}BS(t)dt$$

The integration can be readily computed using the trapezoidal rule, which calculates the area under the prediction curve [48].

ii. Concordance index (C-Index)

The C-index is a discrimination measure that indicates the ability of a model to effectively distinguish between a pair of observations categorized as 'high' and 'low' risk. It is defined as the ratio of concordant pairs to the total comparable pairs [47, 49]. Where a comparable pair means a pair of individuals (say $i$ and $j$) in a dataset such that ${t}_{i}$ and ${t}_{j}$ are its actual event times and $S({t}_{i})$ and $S({t}_{j})$ are their predicted survival times. Now, if a pair $(i, j)$ is such that ${t}_{i}>{t}_{j}$ for which $\left({t}_{i}\right)>$ $S\left({t}_{i}\right)$, this means the actual observed time for ${i}^{th}$ individual is higher than ${j}^{th}$, and the model predicted the same, it is considered as a concordant pair. Otherwise, it is a discordant pair. We can write:

6 $$C-Index=\frac{\#\, of\, Concordant\, Pairs\,}{Total\, Comparable\, Pairs}$$

**Fig. 2:**
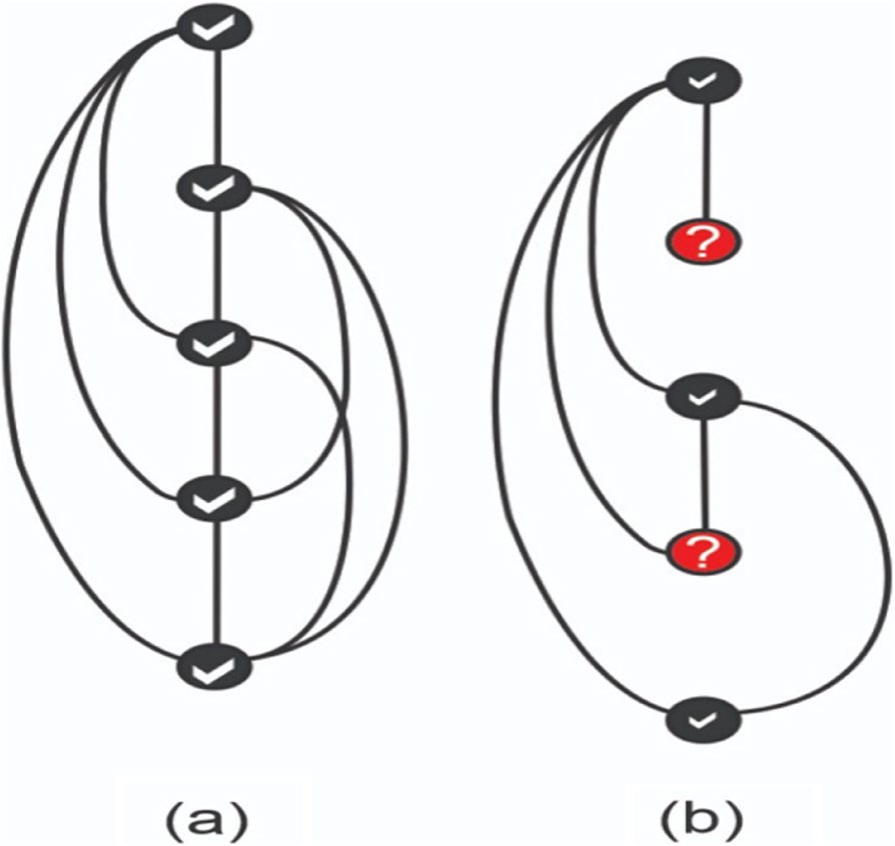
Illustration of making pairs of observations (**a**) with no censored observations and (**b**) with censored observations

To handle censoring when determining comparable pairs, certain rules are followed. For example, a censored instance can only be paired with uncensored instances that occur after it in the dataset. Additionally, a censored instance cannot be paired with either another censored instance or an uncensored instance that occurs after it. This concept is illustrated in Fig. 2, where we have five observations ordered from top to bottom. We have two possible scenarios: (a) All five observations are uncensored, resulting in a total of $^{5}{C}_{2}=10$ pairs. (b) The second and fourth observations are censored, reducing the number of pairs to 6, as per the rule that censored observations cannot be paired with uncensored observations occurring after them.

In survival models that predicts survival time as an output, the C- index is calculated as:

7 $$\widehat{C}= \frac{1}{N}\sum\nolimits_{i:{\delta }_{i}=1}\sum\nolimits_{j:{y}_{i}<{y}_{j}}I[S\left({\widehat{y}}_{j}|{X}_{j}\right)>S\left({\widehat{y}}_{i}|{X}_{i}\right)]$$

Where ‘N’ is the total number of comparable pairs. ‘I[.]’ is the indicator function and S(.) are the estimated survival probabilities from the model. Some survival models do not directly estimate survival probabilities; instead they rather compute hazard ratios, such as Cox PH model. In such cases, the C-index can be computed as:

8 $$\widehat{C}= \frac{1}{N}\sum\nolimits_{i:{\delta }_{i}=1}\sum\nolimits_{j:{y}_{i}<{y}_{j}}I[{X}_{i}\widehat{\beta }> {X}_{j}\widehat{\beta }]$$

Where ‘$\widehat{\beta }$’ represents the estimated parameters calculated from hazard-ratio based models such as Cox, C-index values can range from 0 to 1. A higher value for the C-index indicates better accuracy in terms of separating ‘low’ and ‘high’ risk observations. A C-index equal to 0.5 suggests that the model made random guesses, while a value close to 1 indicates perfect separation, and a value below 0.5 suggests separation in the wrong direction.

iii. Integrated Absolute and Integrated Square Errors (IAE and ISE):

Survival models based on simulated datasets can be evaluated using these two similar methods. This is because the mathematical expression of the survival function *S*(*t*) is typically unknown in practical experiments, but an approximate expression can be obtained using a non-parametric Kaplan–Meier estimation. Using this approximate ‘$S(t)$’, we can obtain the measures IAE and ISE for the real dataset as well. This approximation is available as a built-in function in R package ‘SurvMetrics’ [50]. Thus, if $S(t)$ is the true survival function and $\widehat{S}(t)$ is its estimate, then, the two measures are given as:

$$IAE= {\int }_{t}|S\left(t\right)-\widehat{S}(t)|dt$$

and

$$ISE= {\int }_{t}{(S\left(t\right)-\widehat{S}(t))}^{2}dt$$

The resultant value of these two measures ranges from 0 to infinity, where lower value for a predictive model indicate better predictions. Since the results of both IAE and ISE were quite similar, we therefore reported only IAE in the results section.

## Results and discussion

In this section, we review four tables containing the results of the analysis on 11 high-dimensional survival datasets. The first three tables contain results obtained from the analysis of each of three survival prediction models i.e., Cox Proportional Hazards model, Random Survival Forest and DeepHit employed on five different features selection methods i.e., our proposed method, LASSO, RSF-vs, SCAD and CoxBoost. The results in the table are obtained in similar fashion as explained in the above section. that is, we performed variable selection for each of the 11 datasets and obtained their evaluation metrics values for each dataset corresponding to each feature selection method employed.

Table 2 contains the results of the Cox Proportional Hazards Model when it is employed on different feature selection methods listed in the right-most column of the table. For each of the methods, three performance metrics were computed i.e., Integrated Brier Score, C-index and Integrated Absolute Error. The values of these metrics were recorded in rows corresponding to each variable selection method.

**Table 2:** Performance evaluation metrics for all datasets corresponding to feature selection method followed by Cox PH model

Dataset	Metrics	Proposed	LASSO	RSF-vs	SCAD	CoxBoost
**Breast**	IBS	**0.140535**	0.141248	0.190566	0.141693	0.163642
	CI	**0.867605**	0.855217	0.743754	0.803268	0.864257
	IAE	**0.102332**	0.107944	1.310452	0.108920	0.130010
**WPBC**	IBS	**0.151628**	0.163501	0.169968	0.153057	0.164411
	CI	**0.739101**	0.708902	0.723566	0.707483	0.726452
	IAE	**7.078766**	8.095558	8.889102	8.389040	7.435432
**VDV**	IBS	**0.113642**	0.127571	0.137118	0.174215	0.159096
	CI	**0.864258**	0.820156	0.821653	0.822120	0.821866
	IAE	**0.130010**	0.360298	0.367722	0.359614	0.280468
**Heart FD**	IBS	0.143304	**0.138485**	0.147202	0.139102	0.140283
	CI	0.721703	**0.738974**	0.717210	0.720168	0.738300
	IAE	5.670940	6.019467	6.047454	5.595676	**5.160268**
**MNO**	IBS	**0.190967**	0.233888	0.298705	0.203722	0.195276
	CI	**0.754441**	0.743889	0.742012	0.740748	0.735975
	IAE	**93.82660**	122.7077	111.2272	103.7490	108.0497
**GE1**	IBS	**0.181249**	0.232329	0.223765	0.199710	0.226338
	CI	**0.899909**	0.843480	0.818166	0.808327	0.798787
	IAE	**1.994778**	2.329824	4.226144	2.329394	2.599796
**GE2**	IBS	0.306971	0.186556	0.225465	0.181655	**0.173985**
	CI	0.736727	0.732196	0.699380	**0.766529**	0.738284
	IAE	55.27618	72.01558	108.7328	**49.45919**	75.49166
**GE3**	IBS	**0.139695**	0.149053	0.146137	0.182154	0.158405
	CI	**0.898224**	0.808726	0.719383	0.851940	0.892388
	IAE	**0.246800**	3.830177	0.705477	3.206190	3.872023
**DLBCL**	IBS	0.173810	0.195021	0.517659	0.173366	**0.167596**
	CI	0.804722	**0.810092**	0.706723	0.809995	0.806990
	IAE	0.633490	**0.472370**	1.201690	0.546490	0.546400
**Bone M**	IBS	**0.141842**	0.151039	0.166924	0.148258	0.150969
	CI	**0.767939**	0.749391	0.760324	0.747527	0.749346
	IAE	**18.73366**	59.99366	19.60160	48.45792	45.56484
**NKI**	IBS	**0.134634**	0.142519	0.149063	0.135492	0.145352
	CI	**0.731245**	0.595692	0.668864	0.724220	0.620098
	IAE	**0.621855**	0.816397	0.695028	0.784378	0.735975
**Average**	IBS	**0.165298**	0.169201	0.215688	0.166584	0.167760
	CI	**0.798716**	0.764247	0.738276	0.772939	0.772068
	IAE	**16.755947**	25.158998	23.90952	20.27144	22.715143

The values in bold indicate better performance among all variable selection methods for each dataset. Since three evaluation metrics were considered, we compared all these three metrics for each method corresponding to each dataset. In terms of individual datasets, such as the 'Breast dataset', when features were selected through LASSO and applied to the Cox PH survival model, we observed better predictive performance across all three metrics: IBS, C-index, and IAE. Similar results were obtained from the next two datasets, namely "WPBC" and "VDV", where LASSO feature selection again demonstrated superior performance. However, there appears to be a deviation in the fourth row of the column. In the fourth row of Table 2, we observe the metrics for the 'Heart FD' dataset. Here, we find that the LASSO feature selection method outperformed the others in terms of IBS and C-index. However, it is the CoxBoost FS method that outperformed the others in terms of IAE. This approach allows for a more thorough examination of each dataset, enabling us to assess the performance of each feature selection method relative to others. Each row corresponds to the performance metrics for a single dataset, arranged sequentially. The last row of the Table 2, is named as ‘Average’ which reflects evaluation metrics values averaged across all 11 datasets. This provide us with a comprehensive measure based on which it can be determined which feature selection method outperforms the rest. If we compare the averaged performance across all 11 datasets, it becomes evident that our proposed feature selection method performs exceptionally well across all three metrics.

The comparison of results can be linked to a voting process, where we tally the number of times a method outperforms the others. This involves counting the instances where a feature selection method performs better than the alternatives. By applying the voting criteria for comparison, it becomes evident that in 8 out of 11 datasets, the proposed method surpassed the other methods across all three metrics considered for comparison.

Examining Table 3, which follows a similar format to Table 2, we can compare the outcomes of different feature selection methods in two ways, as discussed earlier. One method involves examining the average value of each metric corresponding to each feature selection method to determine the winner. The other method entails counting the number of times a feature selection method outperforms others across the datasets, which we refer to as "voting". Analyzing the bottom row of the table, we observe that our proposed method surpasses all other four feature selection methods across all comparison metrics, including IBS, C-index, and IAE. Furthermore, when assessing performance based on the number of datasets on which each feature selection method excels, our proposed method outperforms the competition on 9 out of 11 datasets. This indicates that our proposed method demonstrates superior performance according to our "voting" criteria as well. The findings from Table 3 provide substantial evidence to support the assertion that utilizing the proposed method for feature selection, followed by modeling the data using Random Survival Forest, can lead to improved predictive performance in survival analysis.

**Table 3:** Performance evaluation metrics for all datasets corresponding to variable selection method followed by random survival forest

Dataset	Metrics	Proposed	LASSO	RSF-vs	SCAD	CoxBoost
**Breast**	IBS	**0.155878**	0.18544	0.171801	0.177687	0.167882
	CI	**0.895660**	0.882219	0.887316	0.867222	0.878461
	IAE	**0.103923**	0.105296	0.110770	0.112992	0.120003
**WPBC**	IBS	**0.117833**	0.165872	0.166485	0.159112	0.162320
	CI	**0.856080**	0.805282	0.813379	0.779982	0.842492
	IAE	**7.325173**	8.681072	7.416497	7.707982	8.004998
**VDV**	IBS	0.118734	0.127096	0.140105	0.119557	**0.114835**
	CI	0.843267	0.844017	**0.858886**	0.857448	0.846982
	IAE	0.269593	0.274366	0.313366	0.272091	**0.237590**
**Heart FD**	IBS	0.137754	0.135078	0.135004	0.132298	**0.132292**
	CI	**0.855959**	0.843030	0.826422	0.825119	0.841729
	IAE	**6.806459**	7.49524	7.620023	7.116313	8.093140
**MNO**	IBS	**0.206687**	0.233364	0.232527	0.234434	0.2240461
	CI	**0.599092**	0.499804	0.571170	0.500227	0.5002147
	IAE	**95.75326**	97.99927	116.8113	99.13617	101.16250
**GE1**	IBS	**0.191179**	0.21468	0.225003	0.192341	0.240552
	CI	**0.882012**	0.869439	0.875588	0.847204	0.838386
	IAE	**2.199206**	2.391661	2.242796	2.329494	2.248979
**GE2**	IBS	**0.206963**	0.258766	0.211310	0.221533	0.242860
	CI	**0.880097**	0.786075	0.862430	0.799736	0.781516
	IAE	**36.90679**	59.56608	62.63991	57.57227	59.37882
**GE3**	IBS	**0.122479**	0.149154	0.139499	0.151794	0.161327
	CI	**0.885353**	0.824239	0.880720	0.861444	0.878454
	IAE	**3.049394**	3.49487	3.487959	3.347062	3.248837
**DLBCL**	IBS	**0.180658**	0.191903	0.217104	0.191493	0.192339
	CI	**0.874124**	0.848962	0.843127	0.845701	0.849232
	IAE	**0.42595**	0.65403	0.462760	0.714370	0.566990
**Bone M**	IBS	**0.161234**	0.165367	0.162023	0.162869	0.167537
	CI	**0.733525**	0.724380	0.722689	0.723666	0.724853
	IAE	48.83434	50.24475	48.75467	46.90224	**45.147470**
**NKI**	IBS	**0.131234**	0.143904	0.144838	0.145442	0.138554
	CI	0.683935	0.685304	0.691397	0.743216	**0.782807**
	IAE	**0.720843**	0.810597	0.747193	0.733533	0.738320
**Average**	IBS	**0.157330**	0.179148	0.176882	0.171687	0.176777
	CI	**0.817191**	0.782977	0.803011	0.786451	0.796829
	IAE	**18.39954**	21.065203	22.782477	20.540411	20.813422

Table 4, displaying the performance evaluation metric values, follows the same format as the previous tables. It's evident from the results that when the proposed method of variable selection is employed for feature reduction followed by DeepHit-NN as a survival prediction model, 8 out of 11 datasets exhibit lower IBS and IAE values. When examining the C-Index, it's apparent that the proposed method performed even better, with 9 out of 11 datasets showing higher C-index values when the proposed FS method was employed. Additionally, in terms of the average value across all 11 datasets, the proposed method outperformed other FS methods in all three comparison metrics: IBS, IAE, and C-index.

**Table 4:** Performance evaluation metrics for all datasets corresponding to variable selection method followed by DeepHit neural network

Dataset	Metrics	Proposed	LASSO	RSF-vs	SCAD	CoxBoost
**Breast**	IBS	**0.113255**	0.114797	0.125552	0.124476	0.121928
	CI	**0.740659**	0.724126	0.646173	0.696704	0.729015
	IAE	**0.404546**	0.412379	0.456785	0.421668	0.429626
**WPBC**	IBS	**0.208961**	0.230694	0.230451	0.231829	0.231931
	CI	**0.607006**	0.523109	0.533109	0.514974	0.519482
	IAE	**3.178291**	3.599067	3.653415	3.647081	3.730906
**VDV**	IBS	**0.113596**	0.127413	0.120839	0.115618	0.126312
	CI	**0.672175**	0.589376	0.557369	0.630036	0.614054
	IAE	**0.539756**	0.688361	0.59838	0.566711	0.687512
**Heart FD**	IBS	0.161924	0.167742	0.161113	**0.156328**	0.169014
	CI	0.590397	0.531742	**0.604144**	0.598184	0.539913
	IAE	85.10901	83.24036	80.97856	**78.43349**	79.90823
**MNO**	IBS	0.251605	0.253898	0.255841	0.247871	**0.243610**
	CI	**0.579729**	0.554628	0.521921	0.545528	0.574954
	IAE	107.9052	**83.9969**	158.6473	187.4298	151.9972
**GE1**	IBS	**0.254377**	0.311760	0.337485	0.262266	0.283159
	CI	**0.678024**	0.649096	0.606323	0.655086	0.645236
	IAE	**2.364497**	2.506848	2.438306	2.618764	2.415007
**GE2**	IBS	**0.223405**	0.325895	0.348384	0.330284	0.329691
	CI	**0.623580**	0.582128	0.545651	0.604297	0.586916
	IAE	**115.0965**	150.3706	309.2388	293.8419	338.5445
**GE3**	IBS	**0.152755**	0.239756	0.368914	0.330284	0.338077
	CI	**0.638167**	0.620482	0.629033	0.613605	0.622189
	IAE	**3.619512**	3.7688	3.914945	3.951435	3.991879
**DLBCL**	IBS	**0.214506**	0.231646	0.238242	0.330284	0.228449
	CI	**0.599983**	0.596729	0.539118	0.586939	0.592922
	IAE	**1.152548**	1.433048	1.223246	1.397092	1.541776
**Bone M**	IBS	**0.201675**	0.216109	0.227509	0.330284	0.210639
	CI	**0.590841**	0.499735	0.482375	0.502093	0.502661
	IAE	**120.3905**	137.6326	202.5625	209.3423	125.3842
**NKI**	IBS	0.269338	0.269483	**0.268874**	0.330284	0.269779
	CI	0.546614	0.543888	0.559321	0.561748	**0.572084**
	IAE	7.380283	7.427858	7.374283	7.4229	**7.267671**
**Average**	IBS	**0.196854**	0.226290	0.243928	0.253619	0.232054
	CI	**0.624289**	0.583185	0.565867	0.591745	0.590857
	IAE	**40.649149**	43.188801	70.098774	71.73392	65.08168

Table 5 presents the averaged performance evaluation values for five feature selection methods, aligned with each survival model, derived from the average values of each column in Tables 2, 3 and 4. For instance, the IBS value attributed to the 'Proposed' method under the 'Cox PH' model, which is "0.165298", represents the average IBS value obtained from Table 2 under the 'Proposed' method (averaged from the first column). All other values are recorded following the same methodology. This comprehensive approach provides a detailed overview of our entire analysis. The table distinctly illustrates that our proposed feature selection method outperforms other methods across all comparison metrics, including IBS, C-index, and IAE. Referring back to the previous practice of comparison using the voting criteria, it is evident that our proposed method significantly surpasses the performance of other competing feature selection methods.

**Table 5:** Averaged performance metrics across all datasets

Survival Prediction Model	Metrics	Variable Selection Method				
		**Proposed**	**LASSO**	**RSF-vs**	**SCAD**	**CoxBoost**
**COX PH**	IBS	**0.165298**	0.169201	0.215688	0.166584	0.167760
	CI	**0.798716**	0.764247	0.738276	0.772939	0.772068
	IAE	**16.755947**	25.158998	23.90952	20.27144	22.715143
**RSF**	IBS	**0.157330**	0.179148	0.176882	0.171687	0.176777
	CI	**0.817191**	0.782977	0.803011	0.786451	0.796829
	IAE	**18.39954**	21.065203	22.782477	20.540411	20.813422
**DeepHit—NN**	IBS	**0.196854**	0.226290	0.243928	0.253619	0.232054
	CI	**0.624289**	0.583185	0.565867	0.591745	0.590857
	IAE	**40.649149**	43.188801	70.098774	71.73392	65.08168
**Grand Average**	IBS	**0.173161**	0.191546	0.212166	0.197297	0.192197
	CI	**0.746732**	0.710136	0.702385	0.717045	0.719918
	IAE	**25.268212**	29.804334	38.930257	37.515257	36.203415

**Fig. 3:**
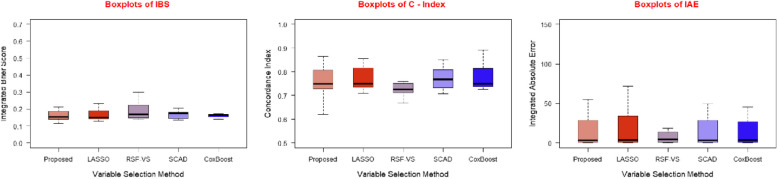
Boxplots of IBS, C-Index and IAE for Cox PH model for five feature selection methods

**Fig. 4:**
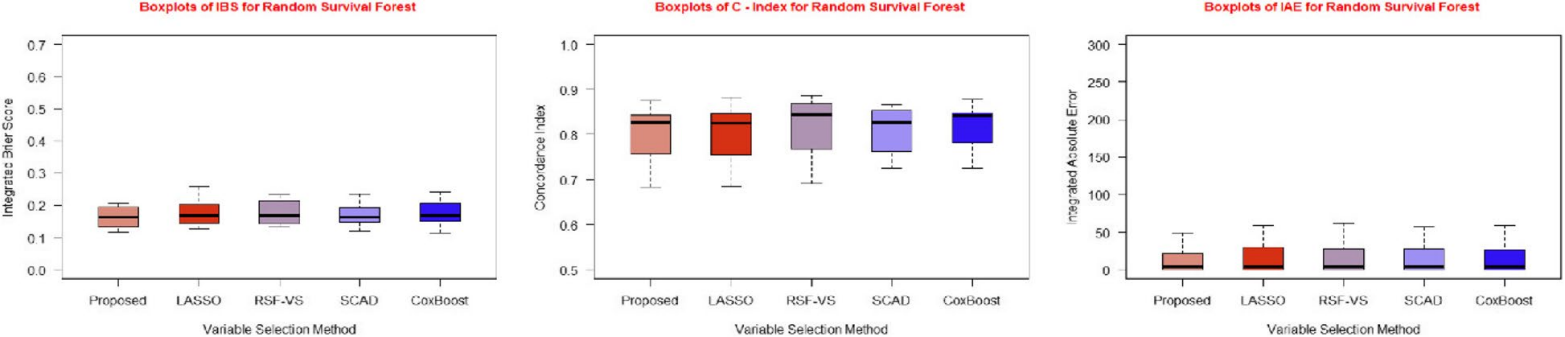
Boxplots of IBS, C-Index and IAE for random survival forest for five feature selection methods

**Fig. 5:**
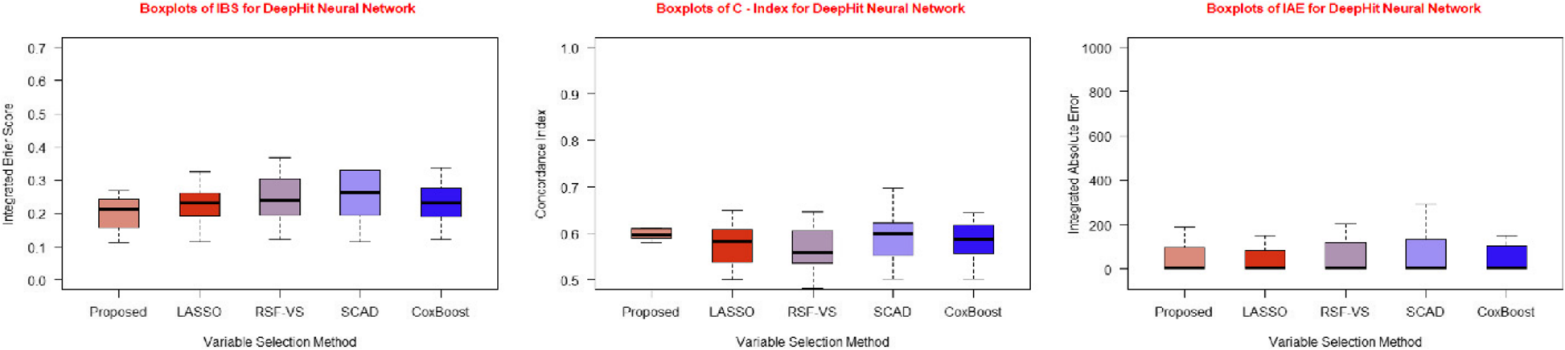
Boxplots of IBS, C Index and IAE for DeepHit for five feature selection methods

The tabulated results are further supported by graphical representations in the form of Figs. 3, 4, and 5, illustrating the performance evaluation metrics for the three different predictive models we employed with each of the five feature selection methods.

## Conclusion

This study aimed to harness a range of existing feature selection techniques to develop a hybrid feature selection (FS) technique that could perform the same task with improved accuracy and reduced margin of error. To assert the accomplishment of the studies objective, we employed a total of five FS methods including the proposed one, along with three different survival models to compute three different performance evaluation metrics namely Integrated Brier Score (IBS), Concordance Index (CI) and Integrated Absolute Error (IAE). The results are presented in both tabular and graphical formats in the results section.

In conclusion, based on the results presented in the preceding section, we have two approaches to determine which method surpasses the others in reducing prediction error and enhancing prediction accuracy. One approach involves comparing how frequently a feature selection (FS) method outperforms others on individual datasets considered in our analysis.

As a rule of thumb, if a feature selection (FS) algorithm performs well on more than two-thirds of the dataset counts, it would be considered ideal. Specifically, in this case, since we are using a total of 11 datasets and employing 5 different FS algorithms, it would be quite rare and ideal for a FS technique to achieve that level of performance consistently across datasets. If a variable selection method performs well on 6–8 datasets, it is considered a satisfactory outcome. We would ideally expect our proposed method to perform at least as well, if not better (on 9–11 datasets).

Another approach to comparing the different feature selection techniques is to assess their average performance across all datasets. Given the detailed discussion of results in the previous section, we now provide a concise conclusion.

In Table 2, the proposed method achieved results above the satisfactory level, outperforming other methods in terms of both the count (8 out of 11 datasets) and averaged results across all three metrics. Moving to Table 3, our proposed method yielded ideal results, outperforming the other methods in terms of both the voting (9 out of 11 datasets) and average comparison. These surprising results align with our expectations, primarily due to the added advantage of using Random Survival Forest as our predictive model. In the case of DeepHit as a prediction model, the proposed technique obtained somewhat similar results to those of Cox PH. In summary, focusing on Table 5, the most comprehensive table, it is evident that the proposed method outperformed the other feature selection methods in terms of all three metrics, whether it is counting/voting or average results. Based on the results provided therein, we conclude that the proposed method improved predictive performance of time-to-event data, especially when the proposed algorithm is employed for dimension reduction and utilized Random Survival Forest for survival prediction.

## Data Availability

No datasets were generated or analysed during the current study.
